# Clinical–Pathological Features and Treatment Outcome of Patients With Hobnail Variant Papillary Thyroid Carcinoma

**DOI:** 10.3389/fendo.2022.842424

**Published:** 2022-03-02

**Authors:** Anello Marcello Poma, Elisabetta Macerola, Agnese Proietti, Paola Vignali, Rebecca Sparavelli, Liborio Torregrossa, Antonio Matrone, Alessio Basolo, Rossella Elisei, Ferruccio Santini, Clara Ugolini

**Affiliations:** ^1^ Department of Surgical, Medical, Molecular Pathology and Critical Area, University of Pisa, Pisa, Italy; ^2^ Department of Clinical and Experimental Medicine, University of Pisa, Pisa, Italy

**Keywords:** hobnail variant, papillary thyroid carcinoma, PTC, treatment outcome, *BRAF*, *TERT*, *RET/PTC*

## Abstract

Papillary thyroid carcinoma (PTC) with hobnail areas above 30% is classified as hobnail variant (HVPTC). Although it is widely accepted that HVPTC has a worse outcome than classical PTC, it is unclear whether PTC with hobnail features below 30% is as aggressive as HVPTC. We gathered the largest mono-institutional series of PTC with hobnail areas and HVPTC to evaluate differences in terms of pathological features of aggressiveness, molecular profile, and treatment outcome. A total of 99 PTC with hobnail features above 5% were retrospectively selected; 34 of them met the criteria for HVPTC (0.4% of all PTC diagnosed at our institution). All tumors showed high rates of extra-thyroidal extension (40.4%), lymph node metastasis (68.1% of patients with lymphadenectomy), and vascular emboli (49.5%), with no differences according to the 30% cutoff. On the other hand, distant metastases were present in HVPTC only (9.4%). Also, advanced age, advanced disease stage, and *TERT* promoter mutation were associated with HVPTC. More than half of the patients with follow-up had structural or biochemical persistence after 1 year from surgery. Structural persistence was significantly more common in patients with HVPTC (37.5% vs. 8.7%), while no differences were observed considering structural and biochemical persistence together. The presence of hobnail features identifies locally aggressive tumors, and, consequently, it should be always acknowledged in the pathological report. However, tumors with more than 30% hobnail areas frequently present *TERT* promoter mutations, advanced disease stage, and structural persistence after radioiodine ablation.

## Introduction

Thyroid carcinoma is the most common endocrine malignancy. The great majority of them are well-differentiated carcinomas, with papillary thyroid carcinoma (PTC) being the most common histotype (1). PTC has generally an excellent outcome following the traditional treatments (i.e., surgery with or without radioiodine ablation) (2). However, some PTC variants are considered more aggressive due to high rates of pathological features of invasion and a troubled clinical management. Aggressive PTC variants include tall cell, columnar, diffuse sclerosing, solid, and hobnail variants (3, 4).

The hobnail variant of papillary thyroid carcinoma (HVPTC) was firstly described by Kakudo et al. in 2004, which noted loss of cell polarity, high nuclear/cytoplasmic ratio, and apical nuclear position that produce a surface bulge and confer the cell a hobnail appearance. The authors attributed these morphologic changes to poor cellular differentiation (5). This suspicion was confirmed by a case series from the Mayo Clinic in 2010, which further described HVPTC and confirmed an aggressive clinical behavior including higher rates of distant metastases, radioiodine refractoriness, and mortality compared with classical variant PTC (CVPTC) (6). Subsequently, other authors have confirmed the poorer outcome of HVPTC patients compared to CVPTC (7–11). Since the latest World Health Organization (WHO) classification of endocrine tumors defines HVPTC by the presence of at least 30% of cells with hobnail features (1), it is not clear whether tumors with lower proportions of hobnail areas deserve to be classified as HVPTC. Some authors have reported that patients with PTC presenting 10% to 30% of hobnail features have similar rates of aggressive pathological features and outcome to that of HVPTC patients (9, 12).

From a molecular point of view, HVPTC presents a very high *BRAF* V600E prevalence (i.e., up to 80%), followed by *RET/PTC* rearrangements. Secondary mutations were also described, especially in *TP53* and *TERT* promoter (1, 6, 8, 12, 13).

Since HVPTC is a rare entity, accounting for approximately 1% of all PTC, the case series presented in literature included a limited number of tumors (14). Larger series are then required to answer the still open questions, especially related to tumors with hobnail features lower than 30%.

Herein, we reported the largest mono-institutional series of PTC with hobnail features above 5% by retrospectively reviewing cases with hobnail areas over the last 6 years. We aimed at investigating differences in terms of clinical–pathological, molecular characteristics, and treatment outcome between HVPTC (i.e., 30% or more hobnail features) and PTC with less than 30% hobnail areas.

## Materials and Methods

### Study Cohort

A retrospective search was conducted in the institutional database by including all the histological reports of PTC containing the term “hobnail” in the period 2015–2020. For each of the retrieved reports, the entire series of hematoxylin and eosin slides were carefully reviewed by three expert pathologists (CU, AgP, and LT), who independently determined the percentage of hobnail areas. The median value was used as final percentage unless a disagreement of 20% or more was present. In these cases, slides were collegially discussed until mutual agreement. Clinical–pathological characteristics of tumors were also collected. A minimal cutoff of 5% hobnail areas was set. Hence, all PTC with at least 5% hobnail areas diagnosed between January 2015 and December 2020 at our institution were included in the study. Cases with foci of poorly differentiated or anaplastic thyroid carcinomas were excluded. All cases were re-staged according to the latest edition of the AJCC/TNM classification of thyroid tumors (15).

In a subset of patients, treatment data including type of surgery and radioiodine ablation were available. Patients were evaluated during the follow-up with regular clinical, biochemical, and imaging procedures, according to the standard of care. At last evaluation, response to the treatment was defined according to the 2015 ATA guidelines (2).

The study fulfills the standards of the Declaration of Helsinki and its subsequent amendments, and was approved by the ethics committee. Written informed consent was signed by each patient.

### Molecular Analyses

For each case, one paraffin block was selected, and four 10-µm-thick sections of tissue were obtained for nucleic acids extraction. After standard deparaffination, tissue enrichment was performed by manually dissecting the areas containing tumor cells. The Qiamp DNA Mini kit and the RNeasy FFPE kit were used for DNA and RNA extraction, respectively (Qiagen, Hilden, Germany).

The presence of mutations in *BRAF* exon 15 and in the promoter of *TERT* was investigated by PCR followed by direct sequencing, as described previously (16). The analysis of *RET/PTC* fusions was conducted in all the *BRAF*-negative cases by using a one-step reverse transcriptional PCR kit, the EasyPGX Ready Thyroid Fusion (Diatech Pharmacogenetics, Jesi, AN, Italy).

### Statistics

Continuous variables were tested for normality distribution by the Shapiro–Wilk test. Variables that did not follow a normal distribution are presented as median and interquartile range (IQR), and were tested by the Mann–Whitney *U* test. Normally distributed variables are presented as mean and standard deviation, and were tested by the Welch *t*-test. For categorical variables, the Chi-square test with Yates’ correction was used; the Fisher exact test was run whenever appropriate. In multivariate setting, a logistic regression was performed, and the Box-Tidwell test was used to check the linear relationship between continuous variables used in the model and the log odds of the outcome. A cutoff of *p* = 0.05 was set as significance level. All analyses were performed in R environment (https://www.r-project.org/, v 4.1.1, last accessed Dec 15, 2021).

## Results

### Clinical–Pathological and Molecular Features

**Figure 1:**
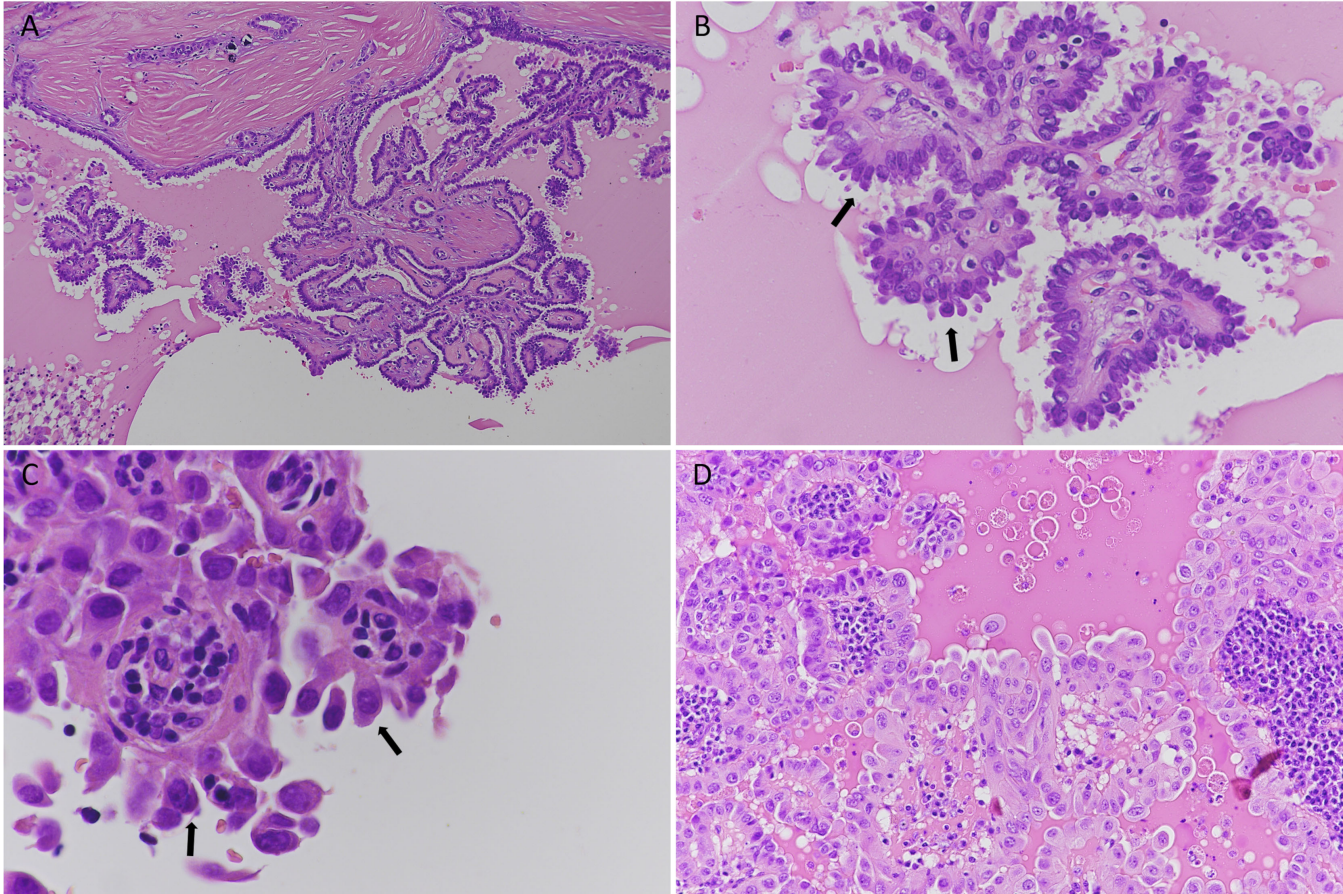
Histological features of hobnail variant papillary thyroid carcinoma (HVPTC); hematoxylin and eosin staining. **(A–C)** A case of HVPTC: **(A)** low magnification (original magnification 10×); intermixed papillary structures lined by neoplastic cells; **(B)** 40× magnification of papillary structures. Hobnail cells are evident (black arrows); **(C)** details of nuclear atypia in hobnail-shaped cells (black arrows, 60×); **(D)** a case of papillary thyroid carcinoma with hobnail features (20×).

From 2015 to 2020, a total of 9,162 PTC were diagnosed at our institution. Among them, 99 PTC (1.1%) had hobnail areas and were included in the study (**Figure 1**). Thirty-four PTC with hobnail areas (0.4% of total PTC) met the criteria of HVPTC (i.e., at least 30% of hobnail areas), while 65 cases were PTC with hobnail areas between 5% and 25%, including 45 classic variants (CVPTC), 15 tall cell variants (TCPTC), 4 solid-trabecular variants (TSVPTC), 1 clear cell variant (CCVPTC).

Overall, the mean age was 49.8 ( ± 15.9) years, the median tumor size was 1.8 cm (IQR 1.3–2.4), and the female-to-male ratio was 1.4. Only 3 PTC (3%) were encapsulated non-invasive. On the contrary, aggressive features were often present, including 40.4% of extra-thyroidal extension (ETE, 6% with T3b or T4 disease), 49.5% of vascular invasion, and 32.3% of lymph node metastases (68.1% of those with lymphadenectomy). *BRAF* mutation (V600E) was present in 69 out of 88 (78.4%) analyzed cases. Six tumors (6.8%) harbored a *RET/PTC* rearrangement, including 4 *RET/PTC1* and 2 *RET/PTC3*. *TERT* promoter mutation was successfully tested in 53 cases, and 8 of them (15.1%) presented the C228T mutation.

Patients with HVPTC were older than those with PTC with hobnail features (*p* = 0.002) and presented more often bilateral tumors (*p* = 0.02) and distant metastases at diagnosis (*p* = 0.03). In addition, HVPTC tumors presented more frequently at advanced stages (*p* = 0.05) and were associated with *TERT* promoter mutation (*p* = 0.02). *BRAF* mutations and *RET* fusions were not associated with hobnail percentage (*p* = 1 and *p* = 0.66 respectively).

Detailed clinical–pathological and mutational data are reported in **Table 1**.

**Table 1:** Clinical–pathological and molecular characteristics of HVPTC and PTC with hobnail features.

Pathological and molecular features		HVPTC (*n* = 34)^§^	PTC with hobnail features (*n* = 65)^§ §^	*p*-value
Gender	male	13 (38.2%)	28 (43.1%)	0.80
Age	years, mean (SD)	56.4 (13.5)	46.3 (16.1)	**0.002**
Size	cm, median (IQR)	1.8 (1.0-2.6)	1.8 (1.3-2.4)	0.93
Extra-thyroidal extension	minimal	8 (25.0%)	26 (40.6%)	0.31
	gross	3 (9.4%)	3 (4.7%)	
Number of emboli	<4	3 (9.4%)	9 (14.1%)	0.87
	≥4	11 (34.9%)	24 (37.5%)	
Multifocal tumor		18 (56.2%)	27 (42.2%)	0.28
Bilateral tumor		16 (50.0%)	15 (23.4%)	**0.02**
Thyroiditis		15 (46.9%)	18 (28.1%)	0.11
Pathologic T stage	T1	21 (65.6%)	39 (60.9%)	0.12
	T2	5 (15.6%)	20 (31.2%)	
	T3-4	6 (18.7%)	5 (7.8%)	
Pathologic N stage*	N1a	5 (38.5%)	12 (35.3%)	1
	N1b	4 (30.8%)	11 (32.3%)	
Extra-nodal extension**		3 (33.3%)	4 (17.4%)	0.37
Distant metastases at diagnosis		3 (9.4%)	0	**0.03**
AJCC TNM stage	stage I	24 (75%)	59 (92.2%)	**0.05**
	stage II	5 (15.6%)	3 (4.7%)	
	stage III–IV	3 (9.4%)	2 (3.1%)	
*BRAF* mutation		24 (77.4%)	45 (78.9%)	1
*RET/PTC* rearrangement		1 (3.2%)	5 (8.8%)	0.66
*TERT* promoter mutation		7 (30.4%)	1 (3.3%)	**0.02**
Lymphadenectomy^§§§^		11 (68.7%)	14 (60.9%)	0.74
RAI ablation^§§§^		13 (81.2%)	20 (86.9%)	0.67
Structural persistence^§§§^		6 (37.5%)	2 (8.7%)	**0.04**
Biochemical persistence^§§§^		4 (25.0%)	8 (34.8%)	0.73

HVPTC, hobnail variant papillary thyroid carcinoma; PTC, papillary thyroid carcinoma; SD, standard deviation; IQR, interquartile range; AJCC, American Joint Committee on Cancer; TNM, tumor node metastases; RAI, radioactive iodine.

^§^2 patients with data not available.

^§§^1 patient with data not available.

^§§§^Cases with follow-up data (i.e., 16 HVPTC and 23 PTC with hobnail features).

*Only cases with lymphadenectomy were considered.

**Only cases with lymph node metastases were considered.

In bold are significant p-values.

### Treatment and Patients’ Outcome

All patients underwent total thyroidectomy, and lymph node dissection was performed on 47 of them (47.5%). According to the latest ATA risk stratification system (2), 17 patients were low risk (17.7%), 68 were intermediate risk (70.8%), and 11 were high risk (11.5%). In three cases, there were no sufficient data to determine the ATA risk category. As expected, PTC with hobnail features were associated with ATA low risk (*p* = 0.005) since patients with HVPTC cannot be classified as low risk (2). For a subset of patients (*n* = 39), follow-up data were available including 16 HVPTC and 23 PTC with hobnail features. The median follow-up was 1 year after surgery. Thirty-three patients (84.6%) received radioiodine ablation, and three of them (7.7% of patients with follow-up) also underwent external beam radiation therapy. No differences between HVPTC and PTC with hobnail features were observed in terms of rate of lymphadenectomy and radioiodine ablation (68.7% vs. 60.9%, *p* = 0.74 and 81.2% vs. 86.9%, *p* = 0.67, respectively). Among patients with follow-up data, 19 (48.7%) had excellent response to therapy (i.e., no clinical, biochemical or structural evidence of disease), eight (20.5%) had structural incomplete response, and 12 (30.8%) had indeterminate response (measurable anti-Tg antibody levels). Structural persistence was associated with HVPTC patients (37.5% vs. 8.7%, *p* = 0.04), while no differences were observed when considering structural or biochemical persistence together (62.5% vs. 43.5%, *p* = 0.40).

We tried to find predictors of structural persistence by a multivariate logistic regression model considering PTC variant (i.e., HVPTC vs. PTC with hobnail features) and the identified confounders (i.e., age, bilaterality, and *TERT* promoter mutation). No strong predictors of structural recurrence were identified, but a trend was observed for HVPTC (*p* = 0.09) and *TERT* promoter mutation (*p* = 0.10). The complete results of multivariate analysis are reported in **Table 2**.

**Table 2:** Multivariate logistic regression model to identify predictors of structural persistence.

Variables	Class	Number SP (*n* = 8)	Number no SP (*n* = 31)	OR (95%CI)	*p*-value
Age				1 (0.93–1.07)	0.91
Bilaterality	No	6	20	1	0.16
	Yes	2	11	0.17 (0.01–1.47)	
Tumor variant	PTC with hobnail features	2	21	1	0.09
	HVPTC	6	10	6.46 (0.85–74.18)	
*TERT* promoter mutation	No	5	29	1	0.10
	Yes	3	2	10 (0.76–295)	

SP, structural persistence; OR, odds ratio; CI, confidence interval; PTC, papillary thyroid carcinoma; HVPTC, hobnail variant papillary thyroid carcinoma.

## Discussion

The hobnail variant papillary thyroid carcinoma (HVPTC) was described for the first time in 2004 (5), and it was referenced in the WHO classification of endocrine tumors only in 2017 (1). Since the first reports, HVPTC was recognized as an aggressive variant (5, 6), and nowadays it is widely accepted that patients with HVPTC have a worse outcome than those with CVPTC (6–11, 17). However, owing to its rarity, there is a need for a wider characterization of tumors with hobnail features, both molecularly and clinically. In particular, the 30% diagnostic cutoff represents a matter of discussion. Tumors with 10% to 30% hobnail features have been associated with poor outcome (9, 12, 14), and a refinement of the diagnostic criteria was already proposed (14). In the present study, we retrospectively gathered the largest series of HVPTC and PTC with 5% to 30% of hobnail areas. HVPTC and PTC with hobnail features did not show evident differences in pathological features of local aggressiveness (i.e., tumor size, extra-thyroidal extension, lymph node metastasis, and vascular emboli). However, distant metastases at diagnosis were present in HVPTC patients only. The latest American Joint Committee on Cancer–tumor node metastasis (AJCC-TNM) staging system determined a remarkable downstaging of thyroid tumors. Accordingly, less than 5% of patients with differentiated thyroid cancer (DTC) should be diagnosed at stage III–IV (18–20). In our series, despite the highly prevalent pathological aggressive features, more than 90% of PTC with hobnail features were diagnosed at stage I, while up to 25% of HVPTC were at disease stage II or higher, including almost 10% of stage III or IV. Therefore, our results confirm that HVPTC patients are expected to have a worse prognosis than those with DTC. It has to be pointed out that the disease stage imbalances herein observed may be due, at least in part, to the older age of patients with HVPTC. Nevertheless, the prevalence of *TERT* promoter mutation observed in HVPTC (i.e., 30%) is in the middle between DTC and ATC, and may reflect a halfway survival rate as well (21). The response to treatment outcome obtained in our series may offer additional perspectives. In our study, although patients underwent similar treatment, structural persistence was more common in those with HVPTC (37.5% vs. 8.7% in PTC with hobnail features), while no significant differences were observed in terms of structural or biochemical persistence considered together (62.5% vs. 43.5%). It was highlighted that in patients with biochemical incomplete response, only a relatively small proportion actually develop structural evidence of disease (22–24), even though subgroups of patients may have a higher risk (25, 26). In addition, the 8.7% of structural persistence observed in PTC with hobnail features is in line with the 7.2% observed in our previous study on CVPTC (27).

This study suffers from some limitations. First, since our institution is a national referral center, many patients that undergo surgery are lost during the follow-up; consequently, the follow-up is short (i.e., approximately 1 year after surgery) and available in a limited number of cases. Hence, we could not evaluate disease recurrence/progression or survival. Second, the sample size is limited, especially for multivariate analysis; as a consequence, the 95% CI of regression analysis is exceptionally wide. On the other hand, this is the largest mono-institutional series of HVPTC and PTC with hobnail features; thus, bias due to subjective morphological interpretation is reduced.

In conclusion, we demonstrated that HVPTC and PTC with hobnail areas showed high rates of locally aggressive features and a relatively low rate of excellent response after standard therapy. However, HVPTC also presented high-risk features such as advanced age, *TERT* promoter mutation, distant metastases, and advanced stage disease. In addition, patients with HVPTC had a high rate of structural persistent disease after radioiodine ablation, which was much higher than in patients with PTC with hobnail areas below 30%. Long-term follow-up data in large monocentric studies will establish whether PTC with hobnail features should be considered as high-risk tumors as well. The presence of any proportion of hobnail features in PTCs should be at least acknowledged in the pathological report.

## Data Availability Statement

The original contributions presented in the study are included in the article/supplementary material. Further inquiries can be directed to the corresponding author.

## Ethics Statement

The studies involving human participants were reviewed and approved by CEAVNO. The patients/participants provided their written informed consent to participate in this study.

## Author Contributions

Conceptualization: AnP, RE, FS, and CU. Methodology: AnP, EM, AgP, PV, RS, LT, AM, AB, and CU. Formal analysis: AnP, EM, and CU. Writing—original draft preparation: AnP, EM, and CU. Writing—review and editing: all authors. Supervision, RE, FS, and CU. All authors contributed to the article and approved the submitted version.

## Funding

This study was funded by the University of Pisa (no specific grant).

## Conflict of Interest

The authors declare that the research was conducted in the absence of any commercial or financial relationships that could be construed as a potential conflict of interest.

## Publisher’s Note

All claims expressed in this article are solely those of the authors and do not necessarily represent those of their affiliated organizations, or those of the publisher, the editors and the reviewers. Any product that may be evaluated in this article, or claim that may be made by its manufacturer, is not guaranteed or endorsed by the publisher.
